# Association between preoperative C-reactive protein to albumin ratio and late arteriovenous fistula dysfunction in hemodialysis patients: a cohort study

**DOI:** 10.1038/s41598-023-38202-w

**Published:** 2023-07-11

**Authors:** Shouliang Hu, Runjing Wang, Tean Ma, Qingfeng Lei, Fanli Yuan, Yong Zhang, Dan Wang, Junzhang Cheng

**Affiliations:** 1 Division of Nephrology, The First Hospital of Yangtze University, No.8, Aviation Road, Shashi District, Jingzhou, Hubei China; 2 Department of Basic Medicine, Xiamen Medical College, Xiamen, China; 3 Division of Nephrology, Jianli County People’s Hospital, Jingzhou, Hubei China; 4 Central Laboratory, The First Hospital of Yangtze University, Jingzhou, Hubei China

**Keywords:** Biomarkers, Nephrology, Risk factors

## Abstract

Arteriovenous fistula (AVF) dysfunction is a critical complication in hemodialysis (HD) patients, with inflammation potentially contributing to its development. This retrospective cohort study aimed to investigate the association between preoperative C-reactive protein to albumin ratio (CAR) and AVF dysfunction in Chinese HD patients. A total of 726 adults with end-stage renal disease who underwent new AVF placement between 2011 and 2019 were included. Multivariable Cox regression and Fine and Gray competing risk models were employed to assess the relationship between CAR and AVF dysfunction, considering death and renal transplantation as competing risks. Among 726 HD patients, 29.2% experienced AVF dysfunction during a median follow-up of 36 months. Adjusted analyses revealed that higher CAR levels were associated with an increased risk of AVF dysfunction, with a 27% higher risk per one-unit increase in CAR. Furthermore, patients with CAR values ≥ 0.153 exhibited a 75% elevated risk compared to those with CAR values < 0.035 (*P* = 0.004). The relationship between CAR and AVF dysfunction varied by the site of internal jugular vein catheter placement (*P* for trend = 0.011). Notably, the Fine and Gray analysis confirmed the association between CAR and AVF dysfunction, with a 31% increased risk per one-unit increase in CAR. The highest CAR tertile remained an independent predictor of AVF dysfunction (HR = 1.77, 95% CI 1.21–2.58, *P* = 0.003). These findings highlight the potential of CAR as a prognostic marker for AVF dysfunction in Chinese HD patients. Clinicians should consider CAR levels and catheter placement site when assessing the risk of AVF dysfunction in this population.

## Introduction

The growing incidence of end-stage renal disease (ESRD) has resulted in a higher utilization of hemodialysis (HD), which is the most prevalent type of kidney replacement therapy. A key challenge in HD is ensuring adequate vascular access, with the arteriovenous fistula (AVF) being the most commonly used approach^1^. However, the longevity of AVF is often threatened by late venous stenosis during the maintenance phase^2^. Primary AVFs have a 1-year and 2-year patency rate of only 64% and 51%, respectively^3^, and vascular access disorders account for a significant portion of hospital admissions and medical expenses for HD patients^4,5^.

Significant progress has been made in understanding the complex mechanisms underlying dysfunction of arteriovenous fistulas (AVFs), which is caused by upstream and downstream events resulting in venous neo-intimal hyperplasia (NIH) and insufficient outward remodeling^6–8^. Although the role of inflammation in AVF dysfunction is well-established, the precise etiology of this phenomenon is still not fully understood^9^. Markers of NIH and inflammation have been associated, however, further research is needed to better understand this relationship^10–14^. It is possible that an initial hemodynamic stress and subsequent inflammation and oxidative stress could trigger a distinct adaptive mechanism that encourages the development of endothelial dysfunction and inflammation. Monitoring strategies based on laboratory tests for inflammation markers may help differentiate stable lesions from those that are likely to thrombose, but further investigation is required in this area^9^.

C-reactive protein (CRP), a widely used clinical marker of inflammation, has been associated with AVF dysfunction^15,16^. In addition, albumin is a protein that is negatively regulated during the acute phase, and hypoalbuminemia has also been linked to AVF dysfunction^17^. CAR, a novel biomarker, has been associated with inflammation, as well as coronary artery disease^18,19^ and peripheral arterial disease^20,21^. The association between CAR and AVF dysfunction in HD patients, if any, is yet to be determined. Hence, our study aimed to explore the potential association between elevated preoperative CAR levels and increased risk of AVF dysfunction in HD patients.

## Materials and methods

### Patients

Between January 1, 2011, and December 31, 2019, a cohort of 1001 end-stage renal disease patients referred for arteriovenous fistula (AVF) formation at the Nephrology Division of Yangtze University’s First Hospital was continuously followed up. A total of 726 eligible patients (72.5%) were included in the study based on the following criteria: (1) mature AVF, (2) adult hemodialysis patients over the age of 18, and (3) preoperative test results before AVF formation. Patients who had not undergone their first surgery, had an immature AVF, had complications following AVF surgery, or had missing CRP and/or Alb data were excluded from the analysis. Information regarding demographics and clinical characteristics were retrieved from the electronic medical record. The minimum follow-up period for each patient was one month until the study's conclusion on December 31, 2020. The final cohort size was not predetermined and was a coincidence. This study (approval number: K20200505) has been granted approval by the Medical Ethics Committee of the First Hospital of Yangtze University. The requirement for obtaining informed consent has been waived. All clinical investigations were carried out in adherence to the principles set forth in the Declaration of Helsinki.

### Evaluation of CAR

The CAR is a value calculated by dividing the preoperative serum CRP level by the Alb level, both of which are measured within 7 days prior to surgery. The CAR is a useful marker for evaluating the patient's general health status before surgery.

### Evaluation of other covariates

The study gathered information on various prognostic factors for AVF dysfunction, drawing on both clinical experience and previous research. The variables included demographic information such as age, sex, diabetes, cardiovascular disease (CVD), drug therapy (ACEI/ARB, statins), and AVF location, as well as lifestyle factors like smoking and IJVC. In addition, various blood test results were analyzed, including levels of Hb, Ca, P, Mg, triglycerides, cholesterol, MPV, RDW, MLR, PLR, NLR, and SIRI.

Blood samples were obtained from participants prior to hemodialysis for analysis. The time between blood collection and sample analysis was within 30 min. Blood counts were determined using the XN-9000 instrument (Sysmex Co., Kobe, Japan). CRP levels were measured using an immunoturbidimetric assay, while Alb levels were determined using a chemical method. Triglycerides and cholesterol were quantified using enzymatic methods. Calcium (Ca), phosphorus (P), and magnesium (Mg) levels were analyzed using colorimetric methods. All these biochemical analyses were performed on the AU5821 automated biochemical analyzer (Beckman Coulter., Ltd.).

### Endpoint assessmen

The primary endpoint of this study is AVF dysfunction, which is characterized by a decrease in vessel diameter greater than 50% or thrombosis, incline of venous pressure, low AVF blood flow and difficult cannulation, requiring surgical intervention or percutaneous transluminal angioplasty.

### Analysis of statistics

From the date of enrollment, each patient in the study cohort was closely monitored until the occurrence of AVF dysfunction or until December 31, 2020, the end of the study. Tertiles were created based on the baseline CAR, dividing the study population into three groups. Normal-distributed continuous variables and categorical variables were compared between groups using ANOVA and χ^2^-test, respectively. To further elucidate the relationship between CAR and AVF dysfunction, Kaplan–Meier plots were constructed, displaying the tertile and extreme tertile of CAR. The Log-rank test was employed to examine the survival differences between groups based on the analyzed plots, as it is a commonly utilized method for this purpose. We utilized a Cox proportional hazard model to explore the potential link between baseline CAR (both continuous and divided into tertiles) and AVF dysfunction. The use of this statistical approach allowed for an assessment of the impact of CAR on the risk of AVF dysfunction while accounting for the effects of potential confounding factors. To account for competing risks, we treated all-cause mortality and kidney transplantation as competing events in our study. Covariates in the Cox regression models were evaluated using the Fine and Gray competing risk model. Results were reported as hazard ratios (HR) with corresponding 95% confidence intervals (CI). These measures provided reliable estimates of the associations observed, quantifying both magnitude and precision. Our comprehensive analysis utilizing these statistical approaches allowed for a thorough evaluation of the relationships between covariates, AVF dysfunction, and the competing risks of mortality and kidney transplantation, ensuring robustness in our findings.

In this study, potential confounders were carefully analyzed individually. Confounders with an estimated change point exceeding 10% or considered clinically significant were included in a multivariate model^18^. The study findings are presented as hazard ratio (HR) and 95% confidence interval (CI), which are the most widely used statistical measures of the association between exposure and outcome. The hazard ratio represents the relative risk of AVF dysfunction associated with a one-unit increase in CAR, while the confidence interval provides information about the precision of the estimate. By examining the relationship between the exposure variable (CAR) and outcome (AVF dysfunction) in the presence of other relevant covariates, the multivariate analysis helps to establish a more accurate estimation of the effect of the exposure variable on the outcome, while controlling for other potential confounding factors.

To handle missing data, this study employed the multivariate multiple imputation with chained formulas technique. This approach, designed to enhance statistical power and reduce potential bias, allows for the imputation of missing data while preserving the relationships between variables^22^. The imputed data were compared to a complete data analysis to ensure the validity of the findings, and further information can be found in the Supplementary Table 3. The analysis was performed using the R software (The R Foundation, version 4.0.5) and Free Statistics 1.8.

### Large-scale language model usage statement

The authors emphasize that the research idea, design, and implementation were the result of their collective wisdom and expertise. The use of ChatGPT was solely for language touch-ups and did not affect the study's content or methodology. The authors collaborated closely to ensure the study's rigor and validity, and the language touch-ups did not alter the scientific integrity or interpretation of the results. Therefore, while ChatGPT enhanced the article's quality and readability, the originality and significance of the research remained the product of the authors' collective effort and expertise.

### Ethical approval

This study (approval number: K20200505) has been granted approval by the Medical Ethics Committee of the First Hospital of Yangtze University. The requirement for obtaining informed consent has been waived. All clinical investigations were carried out in adherence to the principles set forth in the Declaration of Helsinki.

## Results

### Patient characteristics

**Figure 1:**
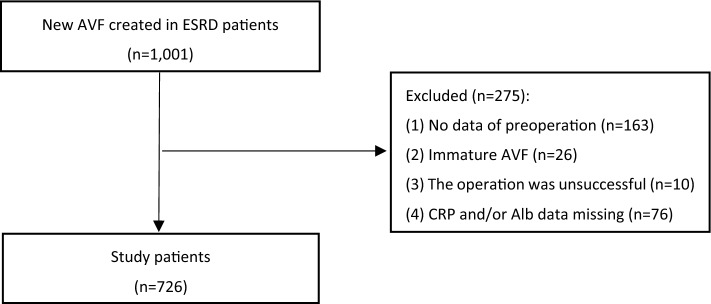
The flowchart of the study. AVF, arteriovenous fistula; ESRD, end-stage renal disease; CRP: C reactive protein; Alb: albumin.

The present study analyzed a total of 726 patients (Fig. 1). The study population had a mean age of 56.0 years with a standard deviation of 12.1, and the majority of the cohort was male, comprising 55.1% of the participants (Table 1). The patients were followed up for a median period of 36 months, ranging from 3 to 119 months. During the follow-up period, 27 patients (3.7%) experienced mortality events, while 4 patients (0.55%) underwent kidney transplantation. Among the study participants, a total of 212 patients (29.2%) experienced permanent arteriovenous fistula (AVF) dysfunction.

**Table 1:** Patient demographic and baseline characteristics.

Variables	Total (n = 726)	CAR prior to AVF created			*p* value
		< 0.035	0.035 ≤ CAR < 0.153	CAR ≥ 0.153	
		(n = 242)	(n = 242)	(n = 242)	
Age, years	56.0 ± 12.1	54.4 ± 12.3	56.4 ± 11.9	57.2 ± 12.0	0.031
Sex, male,n (%)	400 (55.1)	107 (44.2)	129 (53.3)	164 (67.8)	< 0.001
DM, yes, n (%)	126 (17.4)	29 (12.0)	49 (20.2)	48 (19.8)	0.026
IJVC, ipsilateral, n (%)	123 (16.9)	30 (12.4)	45( 18.6)	48 (19.8)	0.065
Smoke, yes, n (%)	127 (17.5)	28 (11.6)	47 (19.4)	52 (21.5)	0.010
CVD, yes, n (%)	53 (7.3)	15 (6.2)	17 (7.0)	21 (8.7)	0.566
AVF location, Forearm, n (%)	703 (96.8)	236 (97.5)	234 (96.7)	233 (96.3)	0.730
ACEI/ARB use, yes, n (%)	703 (96.8)	238 (98.3)	237 (97.9)	228 (94.2)	0.017
Statins use, yes, n (%)	53 (7.3)	14 (5.8)	18 (7.4)	21 (8.7)	0.471
MPV, fL	9.6 ± 1.2	9.7 ± 1.2	9.5 ± 1.2	9.6 ± 1.3	0.435
RDW, fL	14.5 ± 2.1	14.2 ± 1.8	14.6 ± 2.3	14.6 ± 2.0	0.045
Ca, mmol/L	2.0 ± 0.3	2.0 ± 0.2	2.0 ± 0.3	2.0 ± 0.3	0.126
P, mmol/L	1.9 ± 0.6	1.9 ± 0.6	1.9 ± 0.6	1.8 ± 0.6	0.782
Mg, mmol/L	0.9 ± 0.2	1.0 ± 0.2	0.9 ± 0.2	0.9 ± 0.2	< 0.001
Hb, g/L	79.9 ± 18.1	80.1 ± 17.7	80.4 ± 17.7	79.3 ± 19.0	0.782
Alb, g/L	38.2 ± 4.7	39.6 ± 3.9	38.2 ± 4.3	36.7 ± 5.2	< 0.001
SIRI	1.5 (1.0, 2.7)	1.1 (0.7, 1.8)	1.5 (1.0, 2.4)	2.3 (1.5, 3.9)	< 0.001
PLR	163.1 (118.0, 223.2)	143.3 (109.9, 196.8)	161.6 (119.9, 220.1)	185.5 (129.4, 259.7)	< 0.001
MLR	0.3 (0.2, 0.5)	0.3 (0.2, 0.4)	0.3 (0.3, 0.4)	0.4 (0.3, 0.6)	< 0.001
NLR	4.3 (3.0, 6.1)	3.7 (2.7, 4.9)	4.0 (3.0, 5.9)	5.1 (3.7, 7.8)	< 0.001
monocyte, × 109	0.4 (0.3, 0.5)	0.3 (0.2, 0.4)	0.4 (0.3, 0.5)	0.4 (0.3, 0.6)	< 0.001
CRP, ug/L	2.8 (1.0, 9.4)	0.8 (0.5, 1.0)	2.8 (1.9, 4.0)	16.2 (9.4, 32.6)	< 0.001
Triglycerides, mmol/L	1.4 (1.0, 2.0)	1.3 (1.0, 1.7)	1.5 (1.0, 2.1)	1.5 (1.0, 2.1)	0.006
Cholesterol, mmol/L	4.1 (3.4, 4.9)	4.1 (3.4, 4.9)	4.1 (3.5, 5.0)	3.9 (3.3, 4.6)	0.137

MLR: monocyte/lymphocyte ratio, MPV: Mean platelet volume, RDW: Red blood cell distribution width, Hb: Hemoglobin, Alb: albumin, Ca: calcium, P: phosphorus, Mg: magnesium, SIRI: systemic inflammation response index, PLR: platelet-to-lymphocyte ratio, CRP: C reactive protein, IJVC: internal jugular vein catheters, AVF: arteriovenous fistula, CVD: cardiovascular disease, ACEI/ARB: Angiotensin Converting Enzyme Inhibitors/ angiotensin II receptor blockers, CAR: C-reactive protein to albumin ratio.

Comprehensive baseline information for all patients and each tertile of CAR is presented in Table 1. Remarkably, higher CAR levels were generally linked to male gender, IJVC, smoking, ACEI/ARB use, Low levels of hemoglobin and triglycerides, elevated RDW, and increased levels of systemic inflammation indicators including monocyte, SIRI, NLR, PLR, and MLR.

### Primary outcome

**Figure 2:**
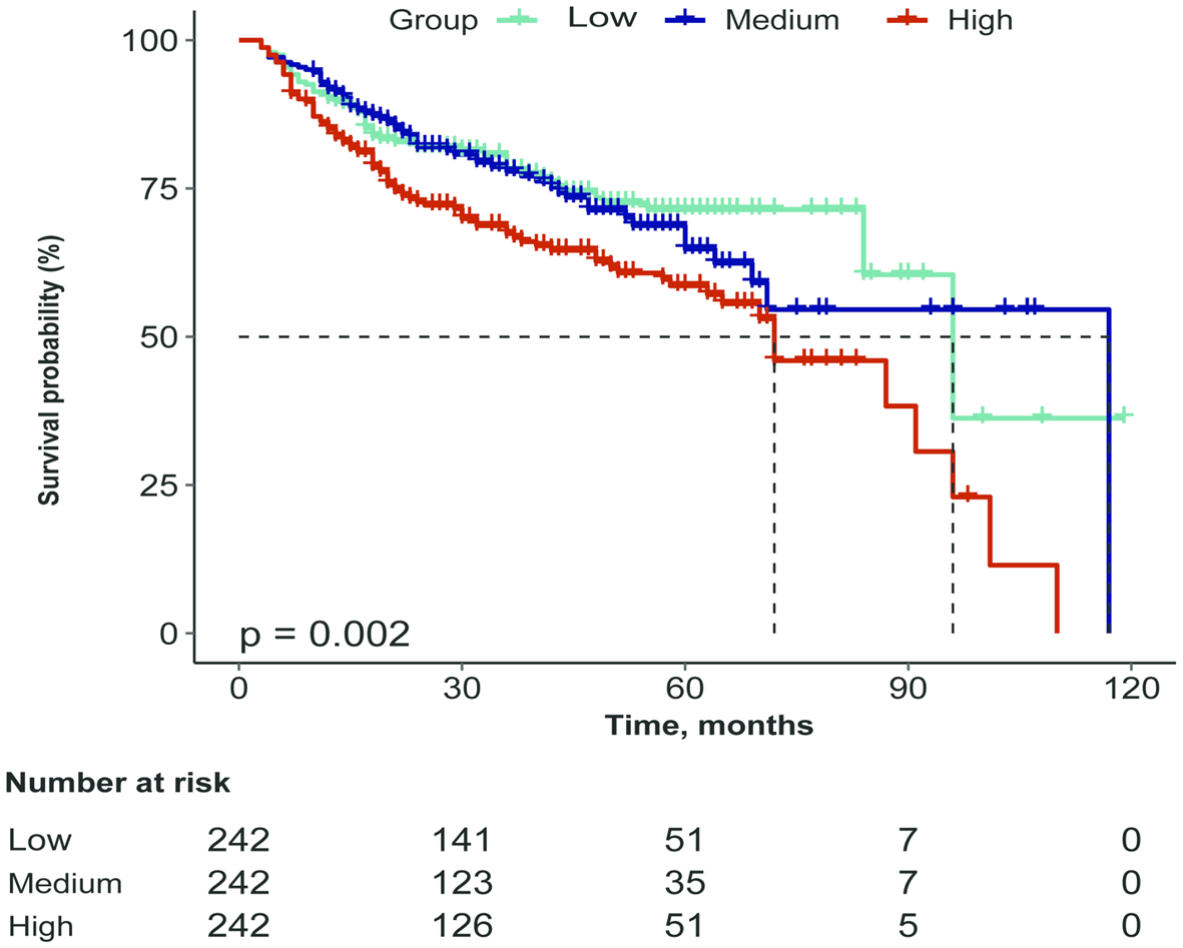
Kaplan–Meier curves of AVF survival in HD patients stratified by CAR tertiles: low < 0.035, medium 0.035–0.153, and high ≥ 0.153. AVF, arteriovenous fistula; HD, hemodialysis; CAR, C-reactive protein to albumin ratio.

The Logrank test demonstrated that there is a noteworthy difference in overall survival between the highest and lowest tertile of CAR, with patients in the highest tertile having a worse survival rate (Logrank test: *P* = 0.002, Fig. 2). However, no significant survival difference was observed between the second and first tertile groups (*P* = 0.611, Fig. 2).

The univariate analysis in this study revealed that several factors, including internal jugular vein catheter placement ipsilateral to the AVF, AVF location, as well as higher CAR and MLR values, are all associated with a higher risk of AVF dysfunction. Furthermore, the proportional increase in AVF dysfunction risk was found to be directly related to the values of CAR and MLR (Supplemental Table 1).

The multivariate analysis revealed that CAR is independently associated with AVF dysfunction, even after adjusting for all potential confounders. Specifically, a 1 unit increase in CAR was linked to a 0.27 times higher risk of AVF dysfunction (HR 1.27; 95% CI 1.04–1.55), indicating that higher preoperative CAR values were dose-dependently associated with an increased hazard of AVF dysfunction. The hazard ratios for AVF dysfunction were 1.04 (95% CI 0.71–1.52) and 1.75 (95% CI 1.20–2.55) for patients with CAR values of 0.035–0.153 and ≥ 0.153, respectively, compared to those with CAR < 0.035. Notably, a significant trend was observed across the different CAR categories (*P* = 0.003; Table 2).

**Table 2:** Relationship between CAR and AVF dysfuncton in different models.

Variable	Model1		Model2		Model3	
	HR 95%CI	*P-*Value	HR 95%CI	*P-*Value	HR 95%CI	*P-*Value
Cox Model						
CAR	1.18 (1.02,1.36)	0.021	1.18 (1.03,1.35)	0.021	1.27 (1.04,1.55)	0.017
CAR (Tertiles)						
Low	1 (Ref)		1 (Ref)		1 (Ref)	
Medium	1.08 (0.76,1.55)	0.524	1.07 (0.74,1.53)	0.730	1.04 (0.71,1.52)	0.832
High	1.68 (1.22,2.33)	0.002	1.83 (1.30,2.58)	0.001	1.75 (1.20,2.55)	0.004
*P* for trend	0.001		0.001		0.003	
Competing risk						
CAR	1.21 (1.07,1.36)	0.002	1.21 (1.09,1.35)	< 0.001	1.31 (1.08,1.58)	0.005
CAR (Tertiles)						
Low	1 (Ref)		1 (Ref)		1 (Ref)	
Medium	0.99 (0.69,1.41)	0.945	1.17 (0.82,1.68)	0.391	1.04 (0.72,1.52)	0.817
High	1.72 (1.24,2.39)	0.001	1.89 (1.32,2.60)	< 0.001	1.77 (1.21,2.58)	0.003
*P* for trend	0.001		< 0.001		0.003	

Model1: Crude Model, Model2 adjusted for age and sex, Model3 adjusted for age, sex, smoke, IJVC, DM, CVD, ACEI/ARB, AVF location, Statin, MPV, RDW, monocyte, Hb, Ca, P, Mg, SIRI, PLR, NLR, MLR, triglycerides, cholesterol. CAR (Tertiles): Low: CAR < 0.035, Medium: 0.035 ≤ CAR < 0.153, High: CAR ≥ 0.153.

HR: hazard ratios, CI: confidence interval, MLR: monocyte/lymphocyte ratio, MPV: Mean platelet volume, RDW: Red blood cell distribution width, Hb: Hemoglobin, Alb: albumin, Ca: calcium, P: phosphorus, Mg: magnesium, SIRI: systemic inflammation response index, PLR: platelet-to-lymphocyte ratio, CRP: C reactive protein, IJVC: internal jugular vein catheters, AVF: arteriovenous fistula, CAR: C-reactive protein to albumin ratio, CVD: cardiovascular disease, ACEI/ARB: Angiotensin Converting Enzyme Inhibitors/angiotensin II receptor blockers.

In the Fine and Gray analysis, where all-cause mortality and kidney transplant were considered as competing risk events, we found a significant association between CAR and AVF dysfunction. For each one-unit increase in CAR, there was a 31% higher risk of AVF dysfunction (HR 1.31; 95% CI 1.08–1.58). Furthermore, the highest CAR tertile remained an independent predictor of AVF dysfunction, with a hazard ratio of 1.77 (95% CI 1.21–2.58, P = 0.003). Importantly, a significant trend was observed across the different CAR categories, further supporting the relationship between CAR and AVF dysfunction (*P* = 0.003; Table 2).

### Sensitivity analyses

Of the 726 patients, those with missing data for triglycerides and cholesterol accounted for 7.2% (52 patients), leaving 674 patients (92.8%) with complete data for all variables in the principal analysis. Imputation was performed to manage the missing data, and Supplemental Table 2 demonstrates that the imputed data shared the same distribution as the observed data of patients with complete information. The results obtained from both the Cox proportional hazards model and the Fine and Gray model, which included only patients with complete information, were consistent with the findings obtained from the multiple imputation datasets. These results are detailed in Supplemental Table 3, providing further confirmation of the associations observed between CAR and AVF dysfunction.

### Subgroup analysis and Interaction testing

**Figure 3:**
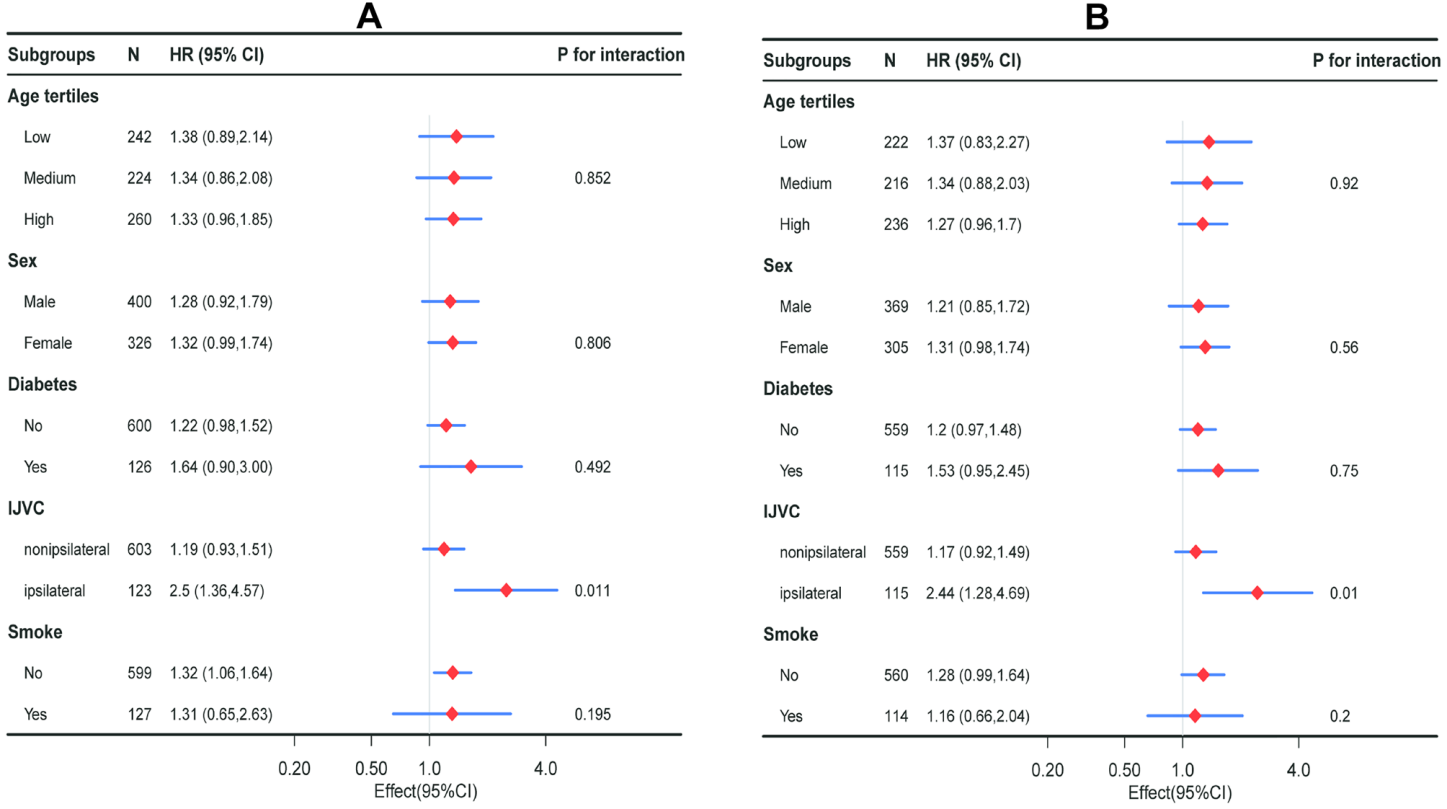
Effect size of CAR on AVF dysfunction in each subgroup. (**A**) Cox Model, (**B**) Competing risk model. Age tertiles: Low 18–50, Medium 51–61, High 62–84. Abbreviations: HR: hazard ratios, CI: confidence interval, IJVC: internal jugular vein catheters, AVF: arteriovenous fistula, CAR: C-reactive protein to albumin ratio.

The study also performed a stratification and interaction analysis to explore potential differences in the relationship between preoperative CAR and AVF dysfunction across different subgroups of the population. The results, displayed in Fig. 3, suggest that the effect of CAR on AVF dysfunction remains stable across various subgroups of the population. The magnitude of the effect of CAR on AVF dysfunction is consistent across subgroups, indicating that the relationship between the two variables is not significantly affected by other factors.

The interaction analysis in the study found that the relationship between preoperative CAR and AVF dysfunction was different depending on the location of internal jugular vein catheter placement relative to the AVF. In the Cox model analysis, patients with internal jugular vein catheter placement on the same side as the AVF exhibited a higher risk of AVF dysfunction (HR: 2.50, 95% CI 1.36–4.57) compared to those with catheter placement on the opposite side (HR: 1.19, 95% CI 0.93–1.51). This observation was further supported by the Competing risk model analysis, which showed a similar pattern with higher HR values for AVF dysfunction among patients with ipsilateral catheter placement (HR: 2.44, 95% CI 1.28–4.69) compared to contralateral placement (HR: 1.17, 95% CI 0.92–1.49). Furthermore, a significant interaction between preoperative CAR and the location of internal jugular vein catheter placement on the incidence of AVF dysfunction was detected (*p*-value for interaction = 0.011 in the Cox model and *p*-value for interaction = 0.01 in the Competing risk model), as illustrated in Fig. 3A and B, respectively.

## Discussion

This study provides compelling evidence that higher levels of preoperative CAR are associated with an increased risk of AVF dysfunction. After accounting for other relevant variables, a dose-dependent relationship between CAR and AVF dysfunction was found, with patients having a CAR value of ≥ 0.153 exhibiting a 1.75-fold higher risk compared to those with CAR < 0.035. Stratified analysis further confirms the robustness of the positive association between CAR and AVF dysfunction. The study also uncovered a significant interaction between CAR and the location of internal jugular vein catheter placement on AVF dysfunction, with patients with high CAR and internal jugular vein catheter placement on the same side as the AVF at a greater risk of AVF dysfunction than those with only one risk factor. These findings provide valuable insights for clinicians and researchers on the potential role of CAR in identifying HD patients at risk of AVF dysfunction.

There have been various attempts in the past few decades to improve the patency rate of AVF. The link between inflammation and AVF dysfunction has been extensively investigated in prior studies^23–25^. During the creation of an AVF, inflammation may arise from either systemic inflammation associated with uremia or local inflammation due to the surgical procedure^24–28^. Systemic inflammation results from the accumulation of waste in the blood due to kidney failure, while the AVF formation process can cause tissue damage, leading to local inflammatory reactions. Both types of inflammation can result in scarring, narrowing, and clotting of the AVF, impairing its function and reducing its patency^29–31^. CRP is an acute-phase protein that rises in response to inflammation or infection in the body. The liver produces CRP in response to signals from cytokines, which are proteins released by immune cells during an inflammatory response. Studies have previously indicated that increased CRP levels could be linked to an elevated risk of vascular access dysfunction in hemodialysis patients^15,16^. Unlike CRP, Alb is the most abundant protein in blood serum, produced by the liver, and its concentration decreases in malnutrition and inflammation^32^. Low preoperative serum albumin levels, indicating reduced serum protein levels, have been suggested to be associated with shorter survival times in patients with AVF and a risk factor for unsuccessful AVF use^33,34^. Previous research has shown that CAR is a more accurate prognostic indicator than either CRP or Alb alone^35^.

The combination of CRP and Alb can provide insight into both inflammation and nutrition, as well as serve as a predictor of patient outcomes^36,37^. Extensive research has been conducted on CAR, an inflammatory biomarker, in patients with vascular diseases, cancer, and infections. Therefore, our study proposes an independent association between preoperative CAR and AVF dysfunction in HD patients. Our study discovered a positive association between preoperative CAR and AVF dysfunction in HD patients. Multivariable Cox regression analysis revealed a significantly increased hazard ratio (HR) of 1.75 for AVF dysfunction associated with higher CAR levels, even after adjusting for all covariates. Subgroup analysis, stratified by factors such as smoking, sex, diabetes, age, and the site of internal jugular vein cannulation, showed that the effect of CAR on AVF dysfunction remained stable across all subgroups. Our study's findings are in agreement with other research that has demonstrated a similar connection between inflammation and AVF dysfunction^30,38,39^. Furthermore, our study observed an intriguing interaction between the placement location of the internal jugular catheter and CAR, which was significant by interaction testing (*p*-value for interaction = 0.011). The relationship between CAR and AVF dysfunction varied depending on whether the internal jugular vein catheter was placed ipsilateral or nonipsilateral to the AVF. In patients with a history of internal jugular vein catheter placement ipsilateral to the AVF, higher preoperative CAR was independently associated with a more significant risk of AVF dysfunction. However, this association was not observed in patients with a history of internal jugular vein catheter placement nonipsilateral to the AVF. If validated, our results imply that patients with a history of internal jugular vein catheter placement ipsilateral to the AVF have a greater likelihood of developing arteriovenous endovascular fistula dysfunction.

The mechanism underlying the interaction between the location of the internal vein jugular catheter placement and CAR is not well understood. We speculate that the mechanism underlying the interaction between the two is due to a combination of several factors. Firstly, histopathological studies have shown that central venous catheterization can cause vascular endothelial injury and central venous stenosis, which affects AVF patency^40^. Hemodialysis catheters (HCs) prior to AVF placement are a known risk factor for complication progression and shortened survival in AVF^41^. Ozpak et al. found that ipsilateral catheter placement significantly shortened cumulative AVF survival and was associated with a higher rate of primary AVF failure than contralateral placement. Thus, the presence of a catheter in close proximity to the AVF may further exacerbate the dysfunction^42^. Secondly, catheters are a proinflammatory stimulus that may increase the risk of infection and inflammation^43^, leading to elevated CRP levels. Sabry et al. found that HD patients with catheters had higher CRP levels, even in the absence of infection, indicating an elevated inflammatory status^44^. The co-existence of ipsilateral catheter placement and high CAR may have a synergistic effect on AVF dysfunction, as both factors independently contribute to inflammation. To sum up, the observed interaction between the location of the internal jugular catheter placement and CAR in relation to AVF dysfunction is likely a result of multiple factors, including vascular endothelial injury, central venous stenosis, inflammation, altered hemodynamics, and potentially others. However, further research is needed to fully understand the underlying mechanism and to develop effective interventions that could help prevent or reduce the risk of AVF dysfunction in hemodialysis patients with prior central venous catheterization. Ultimately, this could lead to improved patient outcomes and a reduction in the morbidity and mortality associated with AVF dysfunction.

There were several strengths of our study. Firstly, continuous patient data collection helps avoid selection bias. Secondly, we treated the target-independent variables as categorical or continuous decreased the probability of analysis errors, thereby increasing the robustness of the results. To ensure the robustness and reliability of our findings, we conducted several sensitivity analyses. These included multiple cox model adjustments, performing a competitive risk model analysis, and employing multiple imputation techniques to address missing data. Importantly, these various analytical approaches consistently yielded similar conclusions, further supporting the validity and strength of our results from multiple perspectives.

Our study, however, has several limitations. First, as a retrospective study, we could not establish a causal relationship between preoperative CAR and AVF dysfunction. Moreover, our sample size limited our ability to adjust for all possible factors related to AVF dysfunction, resulting in potential residual confounding. Second, traditional inflammation indicators such as IL-6 and TNF-α were not measured in our study, as they are more costly and less easily accessible than CRP or Alb. However, our findings suggest that CAR may be a more more economical and practical alternative to these markers in regular postoperative observation of patients undergoing hemodialysis. Thirdly, the generalizability of our findings to other ethnic populations is limited since only Chinese patients were included in our study. Finally, our study focused on AVF dysfunction in mature AVF, and thus, our findings may not be generalizable to immature AVF or cases of restenosis following surgical repair or percutaneous transluminal angioplasty. Although there are some limitations, our study contributes to the expanding research on the link between CAR and AVF dysfunction in HD patients and highlights the necessity for more studies in this field.

In conclusion, our study suggests that preoperative CAR is a significant risk factor for AVF dysfunction in HD patients. Additionally, patients with a combination of high CAR and a history of internal jugular vein catheters placed ipsilateral to the AVF face an even higher risk of AVF dysfunction. However, given the limitations of our retrospective study, future longitudinal prospective studies are needed to establish a causal relationship between CAR, catheter placement, and AVF dysfunction. These findings highlight the importance of routine monitoring of inflammation markers and careful consideration of catheter placement in hemodialysis patients to reduce the risk of AVF dysfunction and its associated complications.

## Supplementary Information

Supplementary Table 1.

Supplementary Table 2.

Supplementary Table 3.

## Data Availability

The data that underlie the results of this study can be made available to interested parties by contacting the corresponding author and submitting a reasonable request.

## Abbreviations

AVF: Arteriovenous fistula
HD: Hemodialysis
ESRD: End stage renal disease
CAR: C-reactive protein to albumin ratio
NIH: Neo-intimal hyperplasia
MLR: Monocyte to lymphocyte ratio
MPV: Mean platelet volume
RDW: Red blood cell distribution width
SIRI: Systemic inflammation response index
PLR: Platelet-to-lymphocyte ratio
IJVC: Internal jugular vein catheters
CVD: Cardiovascular disease
ACEI/ARB: Angiotensin Converting Enzyme Inhibitors/angiotensin II receptor blockers
