# Supplementary material for: Association between preoperative C-reactive protein to albumin ratio and late arteriovenous fistula dysfunction in hemodialysis patients: a cohort study
Source: Sci Rep. 2023 Jul 11;13:11184. doi: 10.1038/s41598-023-38202-w (PMC10336133; doi:10.1038/s41598-023-38202-w)
Supplement: Supplementary file 1 — Supplementary Table 1. [file 41598_2023_38202_MOESM1_ESM.docx]

**Supplemental Table 1**: Univariate analysis for AVF dysfunction

| Covariate | HR(95%CI) | *P*-value |
| --- | --- | --- |
| age,y | 1.0009 (0.9897,1.0122) | 0.881 |
| sex | 1.24 (0.94,1.62) | 0.122 |
| DM | 0.91 (0.63,1.3) | 0.596 |
| IJVC | 0.56 (0.41,0.76) | <0.001 |
| Smoke | 1.02 (0.71,1.47) | 0.904 |
| AVF location | 1.97(1.07,3.62) | 0.029 |
| ACEI/ARB | 1.28(0.60,2.73) | 0.518 |
| CVD | 1.09(0.62,1.91) | 0.764 |
| Statin | 1.15(0.65,2.01) | 0.63 |
| SIRI | 1.0056 (0.9841,1.0276) | 0.636 |
| PLR | 1 (0.9986,1.0014) | 0.975 |
| MLR | 2.01 (1.57,2.57) | <0.001 |
| NLR | 1.0028 (0.9831,1.0229) | 0.789 |
| MPV,fL | 1.01 (0.91,1.12) | 0.848 |
| RDW,fL | 1.06 (0.99,1.13) | 0.073 |
| monocyte,×10^9^/L | 1.3 (0.76,2.22) | 0.336 |
| Hb,g/L | 1.0023 (0.9948,1.0098) | 0.549 |
| CRP,ug/L | 1.0049 (1.0008,1.0089) | 0.018 |
| Alb,g/L | 1.01 (0.98,1.04) | 0.441 |
| triglycerides,mmol/L | 0.9 (0.77,1.05) | 0.172 |
| cholesterol,mmol/L | 0.96 (0.87,1.06) | 0.374 |
| Ca,mmol/L | 1.07 (0.63,1.81) | 0.799 |
| P,mmol/L | 0.93 (0.75,1.16) | 0.536 |
| Mg,mmol/L | 1.8 (0.49,2.81) | 0.714 |
| CAR | 1.18 (1.03,1.36) | 0.019 |

Abbreviations: HR: hazard ratios, CI: confidence interval, MLR: monocyte-to-lymphocyte ratio, MPV: Mean platelet volume, RDW: Red blood cell distribution width, Hb: Hemoglobin, Alb: albumin, Ca: calcium,

P: phosphorus, Mg: magnesium, SIRI: systemic inflammation response index, PLR: platelet-to-lymphocyte ratio,CRP: C reactive protein, IJVC: internal jugular vein catheters, AVF: arteriovenous fistula,

CAR: C-reactive protein to albumin ratio, CVD: cardiovascular disease; ACEI/ARB: Angiotensin Converting Enzyme Inhibitors/ angiotensin II receptor blockers.
