# Supplementary material for: Association between preoperative C-reactive protein to albumin ratio and late arteriovenous fistula dysfunction in hemodialysis patients: a cohort study
Source: Sci Rep. 2023 Jul 11;13:11184. doi: 10.1038/s41598-023-38202-w (PMC10336133; doi:10.1038/s41598-023-38202-w)
Supplement: Supplementary file 2 — Supplementary Table 2. [file 41598_2023_38202_MOESM2_ESM.docx]

**Supplemental Table 2**: Distributions of variables with missing data comparing observed complete case data to results from pooling the datasets with imputed variables from multiple imputation

|  | Number (%)  with missing data | Complete case  Median (IQR) | Multiple imputation  Mean (SD) |
| --- | --- | --- | --- |
| triglycerides | 52(7.2) | 1.7(1.0) | 1.7(1.2) |
| cholesterol | 52(7.2) | 4.3(2.1) | 4.3(2.1) |

Abbreviations: IQR: interquartile range; SD: standard deviation
