# Supplementary material for: Association between preoperative C-reactive protein to albumin ratio and late arteriovenous fistula dysfunction in hemodialysis patients: a cohort study
Source: Sci Rep. 2023 Jul 11;13:11184. doi: 10.1038/s41598-023-38202-w (PMC10336133; doi:10.1038/s41598-023-38202-w)
Supplement: Supplementary file 3 — Supplementary Table 3. [file 41598_2023_38202_MOESM3_ESM.docx]

**Supplemental Table 3:** Complete dataset and multiple imputation datasets unadjusted and confounder adjusted associations of CAR with AVF dysfunction

| Variable | Complete dataset | |  | Multiple imputation datasets | |
| --- | --- | --- | --- | --- | --- |
|  | HR 95%CI | *P-*Value |  | HR 95%CI | *P-*Value |
| **Cox Model** |  |  |  |  |  |
| CAR | 1.27(1.04,1.55) | 0.017 |  | 1.27 (1.05,1.55) | 0.016 |
| CAR(Tertiles) |  |  |  |  |  |
| Low | 1(Ref) |  |  | 1(Ref) |  |
| Medium | 1.04 (0.71,1.52) | 0.832 |  | 1.08 (0.75,1.58) | 0.673 |
| High | 1.75 (1.20,2.55) | 0.004 |  | 1.72 (1.19,2.50) | 0.004 |
| *P* for trend | 0.003 |  |  | 0.003 |  |
| ***Competing risk*** |  |  |  |  |  |
| CAR | 1.31 (1.08,1.58) | 0.005 |  | 1.31 (1.08,1.58) | 0.005 |
| CAR(Tertiles) |  |  |  |  |  |
| Low | 1(Ref) |  |  | 1(Ref) |  |
| Medium | 1.04 (0.72,1.52) | 0.817 |  | 1.07 (0.74,1.55) | 0.704 |
| High | 1.77 (1.21,2.58) | 0.003 |  | 1.67 (1.15,2.42) | 0.007 |
| *P* for trend | 0.003 |  |  | 0.006 |  |

Adjusted for age, sex, smoke, IJVC, DM, CVD, ACEI/ARB, AVF location, Statin, MPV, RDW, monocyte, Hb, Ca, P, Mg, SIRI, PLR, NLR, MLR, triglycerides, cholesterol.

CAR (Tertiles): Low: CAR<0.035, Medium: 0.035≤CAR<0.153, High: CAR≥0.153.

Abbreviations: HR: hazard ratios, CI: confidence interval, MLR: monocyte-to-lymphocyte ratio, MPV: Mean platelet volume, RDW: Red blood cell distribution width, Hb: Hemoglobin,

Ca: calcium, P: phosphorus, Mg: magnesium, SIRI: systemic inflammation response index, PLR: platelet-to-lymphocyte ratio, IJVC: internal jugular vein catheters, AVF: arteriovenous fistula,

CAR: C-reactive protein to albumin ratio, CVD: cardiovascular disease; ACEI/ARB: Angiotensin Converting Enzyme Inhibitors/ angiotensin II receptor blockers.
